# Treatment of intractable isolated bile leakage occurring after right anterior sectionectomy for hepatocellular carcinoma: Right hepatico-jejunostomy

**DOI:** 10.1016/j.ijscr.2024.110125

**Published:** 2024-08-08

**Authors:** Tomomasa Fujii, Takashi Onoe, Naoki Tanimine, Yosuke Shimizu, Hirotaka Tashiro

**Affiliations:** Department of Surgery, Kure Medical Center, National Hospital Organization, 3-1, Aoyama, Kure, Hiroshima 737-0023, Japan

**Keywords:** Case report, Isolated bile leakage, Liver resection, Hepaticojejunostomy, Hepatocellular carcinoma, Surgical management

## Abstract

**Introduction:**

An isolated bile leakage is a relatively rare type of postoperative bile leakage. Most isolated bile leakages require invasive procedures such as surgical approaches.

**Presentation of cases:**

The right hepatic duct was intraoperatively injured during right anterior sectionectomy. Bile leakage occurred postoperatively in the injured bile duct, although the injured bile duct was repaired with suturing and C-tube drainage was performed to decompress the bile duct during hepatectomy. Unfortunately, nonsurgical treatment was not possible. Therefore, bilio-enteric anastomosis between the right hepatic duct and jejunum was ultimately performed because of the small remnant liver volume and poor liver function.

**Discussion:**

Bilio-enteric anastomosis can avoid sacrificing functioning liver parenchyma, but in cases of hepatocellular carcinoma recurrence, transarterial chemoembolization carries a high risk of liver abscess due to cholangitis in patients undergoing enteric revision. Liver resection or bilio-enteric anastomosis should be carefully selected based on clinical data, such as remnant liver volume, liver function, and primary liver disease.

**Conclusion:**

We report a case of isolated bile leakage after anterior sectionectomy for hepatocellular carcinoma that was managed with Roux-en-Y hepaticojejunostomy at the injured right hepatic duct.

## Highlights

• Isolated bile leakage is a relatively rare but intractable.
• Isolated bile leakage needs surgical procedures.
• Hepaticojejunostomy is one of the management for isolated bile leakage.

## 1 Introduction

Bile leakage, which occurs in 2.6–12 % of patients undergoing hepatectomy, is associated with an increased risk of post-hepatectomy liver failure and long hospital stay, leading to a decline in the patient's quality of life and increased mortality [[1], [2], [3], [4]]. Nagano et al. classified bile leakage after hepatectomy into 4 types: type A, minor leakage from a cut surface; type B, leakage caused by insufficient closure of the bile duct stump; type C, leakage from the injured bile duct wall at the exposed bile duct or hilar bile duct; type D, leakage from the distal orifice of the isolated bile duct [5]. Most bile leakage cases are managed using simple drainage. However, some cases that are uncontrolled with drainage require invasive management, such as endoscopic bile drainage and surgical procedures.

We report an intractable case of bile leakage in which the type changed from type C to type D and could not be cured with nonsurgical treatment. The isolated bile leakage was managed by performing a bilio-enteric anastomosis between the injured right hepatic duct and the Roux-en-Y jejunal loop. This work has been reported in line with the SCARE criteria [6].

## 2 Presentation of case

**Fig. 1:**
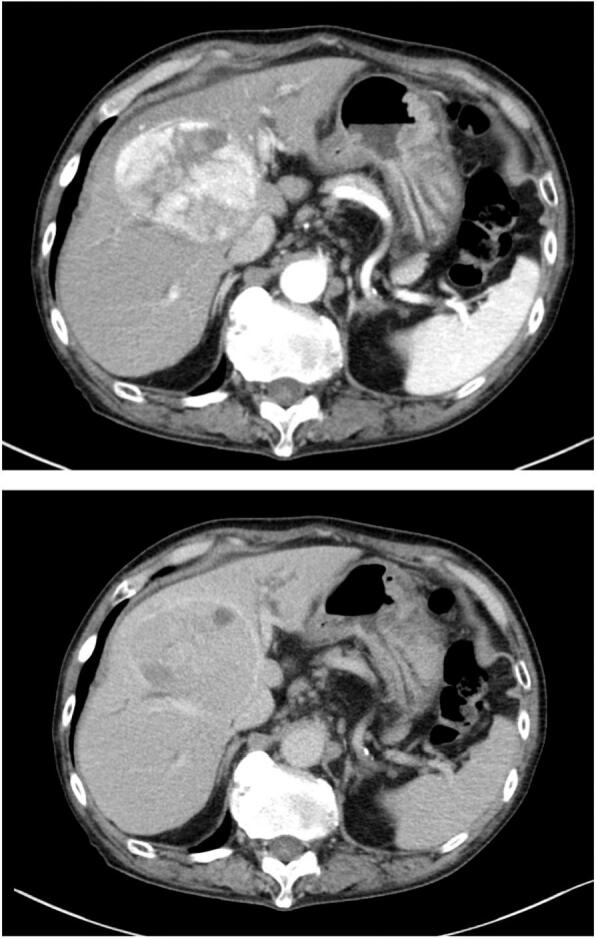
Contrast-enhanced CT shows a 76 mm in diameter tumor. Contrast-enhanced CT reveals a 76 mm diameter tumor with heterogenous enhancement on the arterial phase and a wash-out pattern on the portal phase in the right anterior segment.

A 73-year-old man presented with a liver mass. He was taking medications for hypertension, alcoholic liver disease, and chronic obstructive pulmonary disease. He was diagnosed with hepatocellular carcinoma (HCC) because contrast-enhanced computed tomography (CT) revealed a tumor 76 mm in diameter that showed heterogeneous enhancement in the arterial phase and a wash-out pattern in the portal phase in the right anterior sector of the liver (Fig. 1). Gadolinium-ethoxybenzyl-diethylenetriamine penta-acetic acid-enhanced magnetic resonance imaging (EOB-MRI) showed findings similar to those on CT. No other lymph node or multi-organ metastases were observed on imaging studies. Preoperative examination revealed hepatitis B virus (HBV) infection, and tenofovir alafenamide was prescribed to control the infection.

Preoperative imaging showed that the tumor was compressing the right hepatic vein (RHV) and middle hepatic vein (MHV) and contacting the hepatic hilar plate. His preoperative liver function was classified as Child-Pugh class A; hence, we initially planned right hemihepatectomy with resection of the MHV. However, we considered right hemihepatectomy too invasive because of the low hepatic functional reserve; the indocyanine green retention rate at 15 min (ICG-R15) was 27.1 %, and the adjusted ICG-R15 based on 99mTc-galactosyl serum albumin was 21–25 %.

During surgery, a right anterior sectionectomy was performed. During surgery, the right hepatic duct was injured during the dissection of the adhesion between the HCC and hilar plate, and then the injured bile duct was repaired with continuous suturing using 6/0 polydioxanone; a transcytic duct tube (C-tube) was inserted into the common bile duct through the cystic duct in order to decompress the bile duct. Pathological findings revealed moderately differentiated hepatocellular carcinoma with microvascular invasion into the peripheral portal branch and venous vein, and non-cancerous liver tissues showed liver fibrosis (f4: cirrhosis) according to the general rules for the clinical and pathological study of primary liver cancer [7].

**Fig. 2:**
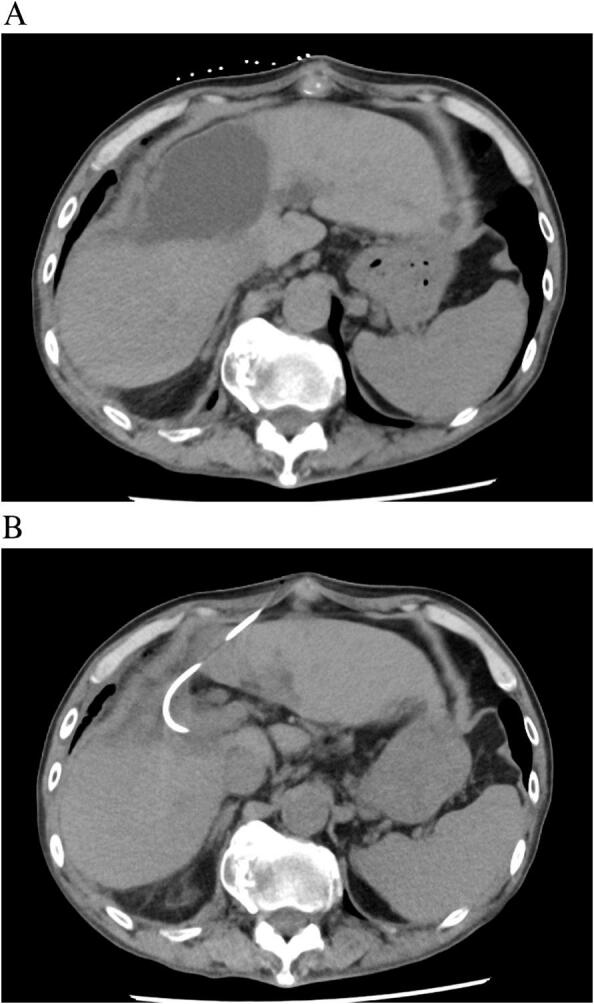
Enlarged fluid collection on the resected liver surface. CT on the 60th postoperative day showed enlargement of fluid collection on the resected liver surface (A). CT-guided percutaneous drainage was performed (B).

**Fig. 3:**
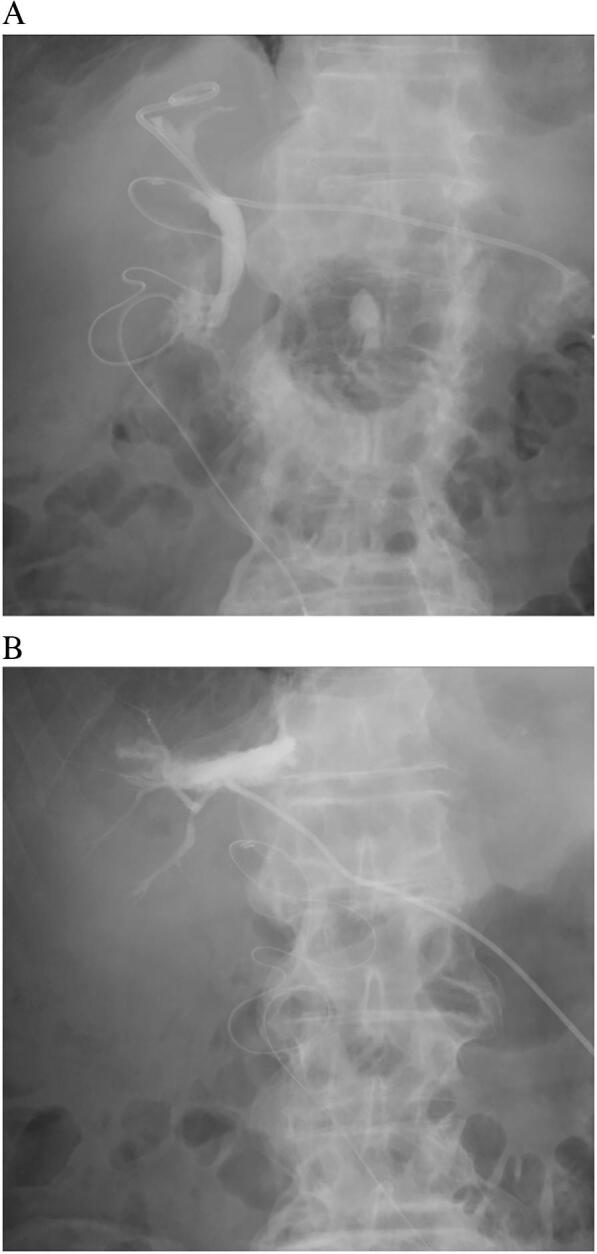
Percutaneous cholangiography via cystic duct tube and fistulography. Percutaneous cholangiography via cystic duct tube indicates no communication between the common bile duct and the right posterior branch (A). Fistulography demonstrates the origin of the fistula from the right posterior branch (B).

**Fig. 4:**
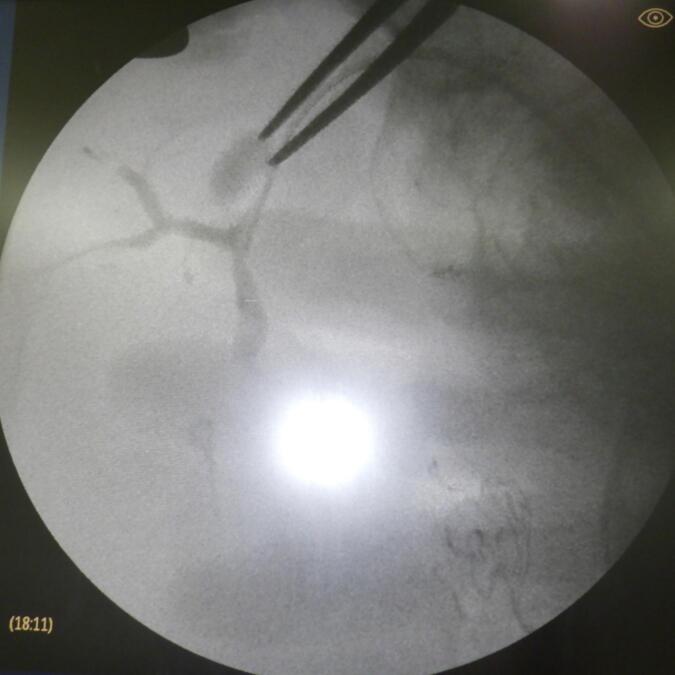
Operative cholangiography. A retrograde transhepatic biliary drainage (RTBD) tube was inserted from the injured site, which was confirmed as the stump of the right posterior bile duct using cholangiography.

**Fig. 5:**
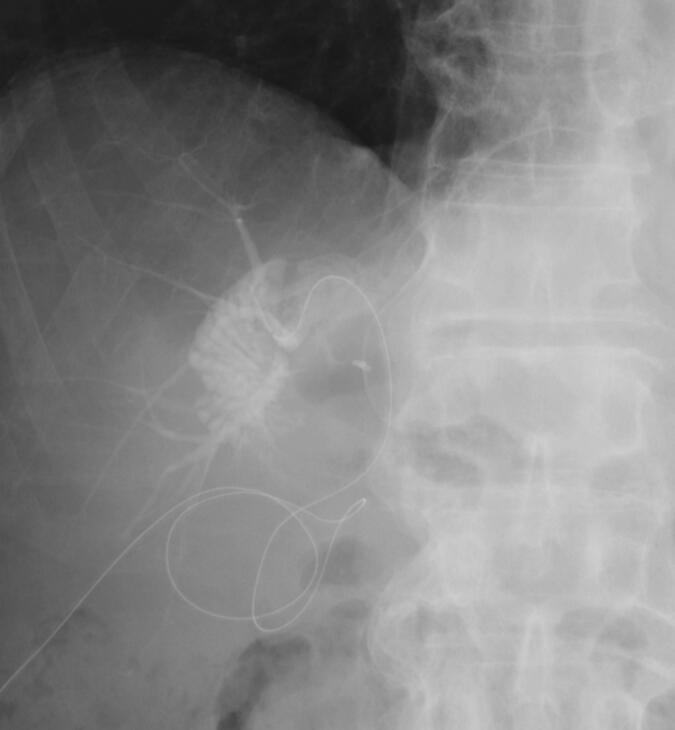
Transjejunal biliary tube cholangiography after six months post-surgery. Percutaneous cholangiography via transjejunal biliary tube after six months shows the absence of bile leakage and no anastomosis stenosis.

On the 7th postoperative day, the patient was febrile (38.5 °C) and peritoneal fluid analysis showed slight elevation of total bilirubin. Contrast-enhanced CT revealed fluid accumulation in the dissection plane of the liver. We continued drainage with a percutaneous drain inserted during the operation and started antibiotic administration. The fluid collection had decreased in size on CT. On the 60th postoperative day, we removed the drainage tube. However, on the 64th postoperative day, EOB-MRI and CT revealed re-enlargement of the fluid collection, and CT-guided percutaneous drainage (Fig. 2). C-tube cholangiography showed appearance of the common bile duct and left hepatic duct but no appearance of the right posterior branch. Percutaneous fistulography revealed an abscess cavity and a right posterior segmental branch of the bile duct (Fig. 3). These findings indicated isolated bile from the posterior branch of the bile duct without communication between the common bile duct and right posterior branch. We attempted initiating communication between the common bile duct and posterior bile duct using endoscopic and percutaneous techniques, but unfortunately, this was impossible. Therefore, liver resection with right posterior sectionectomy was considered an option for repairing bile leakage. However, liver resection was considered too invasive because the future remnant liver volume (remnant left liver) was 27.5 % and the ICGR-15 of the liver remnant was 18.5 %. Therefore, bilio-enteric anastomosis was considered a surgical procedure for repairing bile leakage. A second surgery was performed on the 123rd postoperative day. During the operation, the abscess cavity was easily identified by guiding the percutaneous drainage tube. After careful dissection of the dense adhesions between the hepatic hilar plate and surrounding tissues, the injured right hepatic duct was identified. A retrograde transhepatic biliary drainage (RTBD) tube was inserted from the injured site, which was confirmed as the stump of the right posterior bile duct using cholangiography (Fig. 4). After dissection of 5 mm of the injured bile duct, right hepatico-jejunostomy (end-to-side) was performed without liver resection. The RTBD tube was drained through the afferent limb of the Roux-en-Y. The patient's postoperative course was good. After six months, the RTBD drainage tube was removed after cholangiography, demonstrating no stenosis of the anastomosis (Fig. 5). No bile leakage recurrence was encountered at the one-year follow-up, and there was no recurrence of HCC.

## 3 Discussion

Bile leakage is one of the common complications after hepatectomy. However, type D, isolated bile leakage is a relatively rare complication after hepatectomy, with a frequency ranging from 0.1 to 1 % [[1], [2], [3]]. The isolated bile leakage is intractable and requires various invasive treatments. Shirabe et al. reported that the majority of hepatectomies for isolated bile leakage are major hepatectomies. After right-sided major hepatectomy, isolated bile leakage occurs from the bile duct of segment 1, left segmental bile duct, and the remnant right-side liver [8]. In our case, bile leakage occurred from the injured right hepatic duct after a right anterior sectionectomy. In general, hepatectomies such as central bisectionectomy, anterior sectionectomy, and segmentectomy of segments 1 and 4, which expose the hilar plate, are independent risk factors for bile leakage [4]. Our case was thought to correspond to type C, which is leakage from the injured bile duct at the exposed bile duct or hilar bile duct, since the right hepatic duct was injured during right anterior sectionectomy. However, the injured bile duct was thought to be stenotic with fibrosis during healing. Consequently, isolated bile leakage (Type D) may have occurred because of an increase in biliary internal pressure resulting from biliary stricture of the central side of the injured right hepatic duct. Sakamoto et al. also reported four cases of bile leakage with strictures of the right hepatic duct, all of which had undergone anterior sectionectomy [9].

Transcystic duct tube drainage (C-tube drainage) has been reported to be useful for preventing post-hepatectomy bile leakage by decompressing the internal pressure of the bile duct during surgery. Hotta et al. observed a bile leakage rate of 3.6 % in patients with C-tube drainage and 26.3 % in those without drainage [10]. In our case, C-tube drainage was placed during anterior sectionectomy, but was not effective. Nanashima et al. also reported that the bile leakage rate did not differ between the C-tube and non-C-tube groups [11]. C-tube drainage may not be effective in preventing bile leakage in patients with complicated bile duct injury. Even if a C-tube is inserted through the cystic duct, the tip of the tube enters the common bile duct, thus precluding prophylactic drainage of the injured right (or left) bile duct. If the right (or left) bile duct is injured, the C-tube is inserted through the opening of the injured bile duct to bridge the distal and proximal portions, and the applicator-attached end of the C-tube is withdrawn from the common bile duct to outside the bile duct, after which the injured bile duct is closed by interrupted sutures. The C-tube is then secured by using a 5-0 PDS and guided out of the body. This technique may be very useful to decompress the proximal portion of the bile duct. This technique is based on the insertion of a bile duct stent tube at the anastomotic site of bile duct to duct anastomosis in living donor liver transplantation.

Various treatments have been proposed for managing isolated bile leakage after hepatectomy. These methods depend on the remnant liver function and the amount of bile leakage. Non-surgical approaches, including percutaneous or endoscopic drainage, are only suitable for minor bile leakages. Tanaka et al. proposed a minimally invasive treatment with ethanol or fibrin glue injection as the first-line treatment [12]. However, in cases of intractable or extensive bile leakage, invasive interventions like percutaneous transhepatic portal vein embolization (PTPE) [13], liver resection, and bilioenteric anastomosis become necessary.

In the present case, we attempted endoscopic and percutaneous stenting of the damaged bile duct; however, this was impossible. Sakamoto et al. reported that two of four cases of bile leakage from the right hepatic duct were manageable by placement of a stent beyond the stricture [9]. The following events were considered as causes of unsuccessful non-surgical procedures: 1) stricture of the right hepatic duct as mentioned above, and 2) deformity of the right hepatic duct (protrusion of the right hepatic duct into the caudal side), which was induced by a defect in the right anterior section and hypertrophy of the right posterior section. Consequently, communication between the common bile duct and the right hepatic duct could not be achieved by endoscopic and percutaneous techniques.

Next, ethanol or fibrin glue injection therapy was not indicated in our case because the liver volume of the posterior segment was large, and the amount of bile was high. PTPE of the posterior segment is also considered too invasive, and this procedure should not be chosen to avoid liver failure because of the future remnant volume of the left liver (27.5 %) and liver fibrosis (F4).

Surgical procedures are an alternative strategy in cases where non-surgical procedures are ineffective. Honore et al. reported two patients with isolated bile leakage, two of whom were treated with hepatectomy. Liver resection is a difficult but definitive treatment for cases where nonsurgical interventions were unsuccessful [14]. Patrono et al. reported two cases of excluded segmental bile duct leakage occurring after hepatic resection managed by Roux-en-Y hepaticojejunostomy [15]. They reported that this procedure can avoid sacrificing the functioning hepatic parenchyma.

In our case, hepatic resection by posterior sectionectomy was difficult due to the small future remnant liver volume and poor liver functional reserve. For these reasons, bilioenteric anastomosis was performed. In bilioenteric anastomosis, it is sometimes difficult to clearly dissect and identify the origin of the bile leak in the biliary duct stump. Therefore, a new anastomosis (fistula-jejunostomy) between the jejunum and bile fistula is sometimes created using a drainage catheter. In these cases, incomplete anastomosis may lead to post-operative bile leakage [9]. Bilioenteric anastomosis also involves the risk of biliary tract infection, whereas liver resection, which removes functioning hepatic parenchyma, carries a risk of post-operative liver failure. Liver abscess is likely to occur after transarterial chemoembolization for HCC recurrence in patients with enterobiliary anastomosis. Bilioenteric anastomosis may be avoided in patients with HCC with a high risk of recurrence. Liver resection or bilioenteric anastomosis should be carefully selected based on clinical data, such as future remnant liver volume, liver function, and primary liver disease, as indications for hepatectomy. Moreover, because adhesions resulting from inflammation following bile leak make surgical procedures difficult, a second operation should be performed by experienced hepatobiliary surgeons.

## 4 Conclusion

Here, we report a case of isolated bile leakage after right anterior sectionectomy managed with a right hepaticojejunostomy. Surgical treatments are essential and effective for bile leaks in which non-surgical interventions are unsuccessful. Bilio-enteric anastomosis is a useful surgical procedure for the treatment of isolated biliary leakage in patients with poor liver function.

## Funding

This research did not receive any specific grant from funding agencies in the public, commercial, or not-for-profit sectors.

## Consent for publication

Written informed consent was obtained from the patient for publication and any accompanying images. A copy of the written consent is available for review by the Editor-in-Chief of this journal on request.

## Declaration of competing interest

There are no conflicts of interest to declare.
